# A systematic review of the clinical features of pneumonia in children aged 5-9 years: Implications for guidelines and research

**DOI:** 10.7189/jogh.12.10002

**Published:** 2022-03-26

**Authors:** Priya M Kevat, Melinda Morpeth, Hamish Graham, Amy Z Gray

**Affiliations:** 1 Murdoch Children’s Research Institute, Melbourne, Victoria, Australia; 2 University of Melbourne, Melbourne, Victoria, Australia; 3 Royal Children’s Hospital Melbourne, Melbourne, Victoria, Australia

## Abstract

**Background:**

Childhood pneumonia presents a large global burden, though most data and guidelines focus on children less than 5 years old. Less information is available about the clinical presentation of pneumonia in children 5-9 years of age. Appropriate diagnostic and treatment algorithms may differ from those applied to younger children. This systematic literature review aimed to identify clinical features of pneumonia in children aged 5-9 years, with a focus on delineation from other age groups and comparison with existing WHO guidance for pneumonia in children less than 5 years old.

**Methods:**

We searched MEDLINE, EMBASE and PubMed databases for publications that described clinical features of pneumonia in children 5-9 years old, from any country with no date restriction in English. The quality of included studies was evaluated using a modified Effective Public Health Project Practice (EPHPP) tool. Data relating to research context, study type, clinical features of pneumonia and comparisons with children less than 5 years old were extracted. For each clinical feature of pneumonia, we described mean percentage (95% confidence interval) of participants with this finding in terms of aetiology (all cause vs *Mycoplasma pneumoniae*), and method of diagnosis (radiological vs clinical).

**Results:**

We included 15 publications, eight addressing all-cause pneumonia and seven addressing *Mycoplasma pneumoniae*. Cough and fever were common in children aged 5-9 years with pneumonia. Tachypnoea was documented in around half of patients. Dyspnoea/difficulty breathing and chest indrawing were present in approximately half of all-cause pneumonia cases, with no data on indrawing in the outpatient setting. Chest and abdominal pain were documented in around one third of cases of all-cause pneumonia, based on limited numbers. In addition to markers of pneumonia severity used in children <5 years, pallor has been identified as being associated with poorer outcomes alongside comorbidities and nutritional status.

**Conclusions:**

Quality research exploring clinical features of pneumonia, treatment and outcomes in children aged 5-9 years using consistent inclusion criteria, definitions of features and age ranges are urgently needed to better inform practice and guidelines. Based on limited data fever and cough are common in this age group, but tachypnoea cannot be relied on for diagnosis. While waiting for better evidence, broader attention to features such as chest and abdominal pain, the role of chest radiographs for diagnosis in the absence of symptoms such as tachypnoea, and risk factors which may influence patient disposition (chest indrawing, pallor, nutritional status) warrant consideration by clinicians.

**Protocol registration:**

PROSPERO: CRD42020213837.

Childhood pneumonia is responsible for a large mortality burden globally however most guidelines for low resource settings are focused on pneumonia in children less than 5 years old [1,2]. Focus on young children has been justified by the fact that more than 90% of childhood pneumonia deaths occur in young children less than 5 years of age [3]. Yet pneumonia is also important for older children. Global Burden of Disease estimates suggest that pneumonia accounts for around 7% of deaths in children aged 5-9 years [3].

While children aged 5-9 years are generally regarded as at lower risk for pneumonia and pneumonia death, the risk may still be substantial in certain contexts or patient cohorts (for example, children with chronic health conditions or disability). Appropriate diagnostic and treatment algorithms may differ from those applied to younger children and this group has not been addressed in previous guidelines.

The aim of this review was to describe the available evidence for clinical features of pneumonia in children aged 5-9 years in community, primary care, or hospital settings, with a focus on delineation from other age groups and comparison with existing WHO guidance for pneumonia in young children.

## METHODS

The protocol for this study was registered on PROSPERO, the international prospective register of systematic reviews (registration number CRD42020213837). We searched MEDLINE via Ovid, EMBASE via Ovid and PubMed in August 2020 using key search terms including synonyms for pneumonia, ages 5-9 years, and clinical findings or diagnosis (example in Appendix S1 in the **Online Supplementary Document**). No date restriction was applied. We did not restrict by location of study but for practical reasons we restricted the search to studies available in English language.

We included studies that contained original data on the clinical features of pneumonia among children aged 5-9 years, published in English language. We excluded case reports, small case series (<10 participants), conference abstracts, or those in which data relating to children aged 5-9 years was not meaningfully disaggregated.

PK completed initial title and abstract screening. Full-text screening was completed by three reviewers (PK, MM, AG), with each article screened by two of these reviewers (PK, MM, AG) and any conflicts resolved by the majority opinion from the third remaining reviewer (PK, MM, AG). Reference lists of included articles were searched to identify additional relevant studies missed from the search.

We extracted data from included studies with a standardised data extraction tool. Information extracted included: year of publication, study details, inclusion and exclusion criteria, pneumonia diagnostic/case definition criteria, aetiological agent(s), participant characteristics (including socioeconomic status), presence of comorbid conditions, respiratory and extra-pulmonary clinical features, chest radiograph findings, treatment received, and outcomes, with comparison to the under 5 years age group wherever possible. Data extraction was completed by two reviewers (PK, MM), with data from each article extracted by one of these reviewers (PK, MM) and the extracted information checked by the second reviewer (PK, MM). Any conflicts were resolved by the majority opinion from a third reviewer (AG).

We separated data from studies describing pneumonia of any aetiology (all-cause pneumonia) and studies describing pneumonia attributed to *Mycoplasma pneumoniae*, given that several studies addressed *Mycoplasma pneumoniae* specifically. For each clinical feature, we described the number and percentage of patients who were documented to have the feature in each study. Using aggregated data of all studies which included each clinical feature we calculated the mean percentage and 95% confidence interval according to the cause of pneumonia (all-cause and attributable to *Mycoplasma pneumoniae*) and the method of diagnosis (radiological or clinical). If studies stipulated their inclusion criteria as a clinical diagnosis with or without radiological diagnosis, they were included in the studies based on clinical diagnosis for analysis (as we were unable to identify which participants had a radiograph performed). Due to the relatively weak quality of the studies identified and the variable nature of the data from the studies we did not perform any additional statistical analysis, to avoid over-interpretation of the data available.

We used the EPHPP tool to evaluate the risk of bias in included studies [4]. This tool was modified to assess the study designs included (Table S1 in the **Online Supplementary Document**). Application of the EPHPP tool required separate evaluation and consensus between two reviewers (PK, MM).

The Preferred Reporting Items for Systematic Reviews and Meta-Analyses (PRISMA) 2020 statement was followed, with a checklist completed (Table S2 in the **Online Supplementary Document**) [5].

## RESULTS

**Figure 1:**
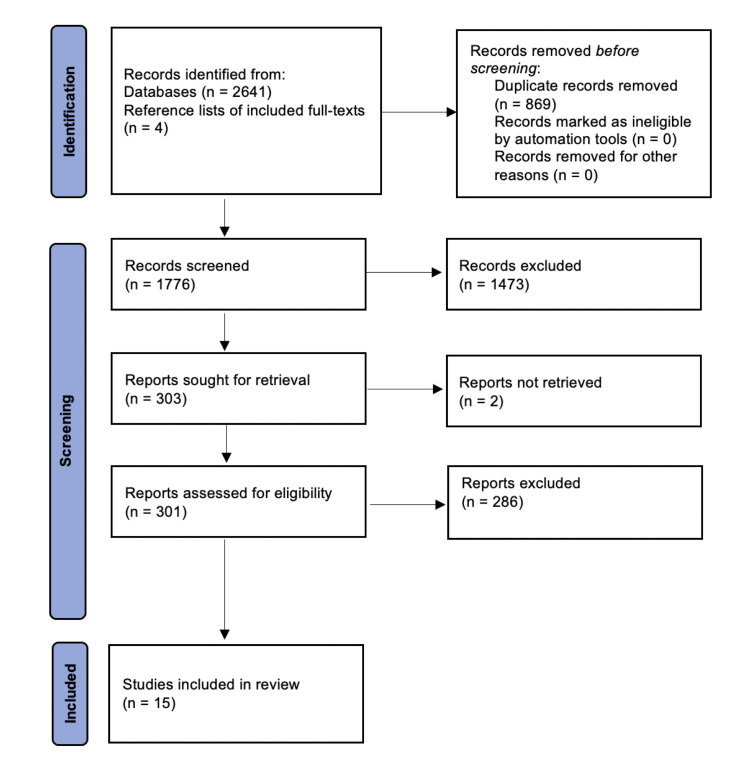
Preferred Reporting Items for Systematic Reviews and Meta-Analyses (PRISMA) flow diagram.

A total of 2641 references were retrieved, and an additional four relevant publications were identified through reference list screening (**Figure 1**). After duplicates were removed, 1776 references were screened, and 301 proceeded to full-text review. Two articles were excluded as the full text was unavailable, after authors were contacted twice to request them. Fifteen studies were included in qualitative synthesis after inclusion and exclusion criteria were applied.

### Study descriptions

Studies had variable methods to identify patients with pneumonia. Seven of the 15 studies included children with radiologically confirmed pneumonia (two of these requiring clinical features in addition) and eight of the 15 studies were based on clinical diagnosis with or without a radiograph. The heterogeny in diagnostic methods was significant. For example, one study based on radiological diagnosis only included patients with obvious chest indrawing. Furthermore, of those based on clinical diagnosis, three studies included children with or without a radiograph being performed, three required clinician diagnosis alone, and two studies were of *Mycoplasma pneumoniae* positive patients that described clinical features and/or chest radiograph changes consistent with pneumonia. Eight studies addressed all-cause pneumonia (**Table 1**) whilst seven discussed pneumonia attributable to *Mycoplasma pneumonia*e, based on a variety of diagnostic assays (**Table 2**). Three out of the 15 studies, Macpherson et al [12], Salih et al [13] and Forgie et al [11], were from low or lower-middle income settings. Twelve studies described inpatients only, one study by Harris et al [9] was of outpatients, and two studies by Korppi et al [8] and Othman et al [19] included a combination of inpatients and outpatients.

**Table 1:** Clinical features described in children aged 5-9 y diagnosed with pneumonia of any aetiology (all-cause pneumonia)

Authors, year	Study location, design & population	Patient numbers	Respiratory symptoms/signs	Extra-pulmonary symptoms/signs	Chest x-ray findings	Comparison with <5 y age group	EPHPP global rating score
**Studies based on radiological pneumonia diagnosis:**							
Crocker et al, 2012 [6]	South Wales, UK	79 total	Most relevant data not disaggregated by age	Most relevant data not disaggregated by age	For 3-16 y:	Less common in <3 y compared with 3-16 y:	Weak
	Questionnaire + interview with prospectively recruited carers of inpatients with radiographic CAP or empyema (excluding chronic conditions)	43 (54%) 3-16 y		For 3-16 y:	12/43 (28%) with pleural effusion or empyema	Pain in torso (symptom volunteered as unusual or concerning): 2/36 (5.6%) vs 15/43 (34.9%) (*P* < 0.01)	
				Symptoms reported when asked about presence or absence by the interviewer:			
				36/43 (84%) pain in torso (usually chest or abdomen) including all 12 with pleural effusion or empyema		NB: The number of children 3-16 y with pain in the torso was 36/43 (84%) when the interviewer asked about the presence or absence of the symptom as a closed question.	
				23/43 (54%) headache			
				12/43 (28%) general aching			
				15/43 (35%) back pain			
				10/43 (23%) side pain			
				6/43 (14%) shoulder pain			
				14/43 (33%) pain at other sites (legs/neck/arm)			
Juvén et al, 2003 [7]	Turku, Finland	254 total	For children ≥5 y:	For children ≥5 y:	Not specified	More common in <2 y compared with 2-4 y and children ≥5 y:*	Weak
	Prospective study of inpatients with radiologically confirmed CAP (information regarding comorbid conditions not specified)	62 (24%) children ≥5 y	50/62 (81%) cough	60/62 (97%) fever >37.5°C		Rhinorrhoea: 58% vs 41% and 39% respectively	
			24/62 (39%) rhinorrhoea	~ 12/62 (20%) poor appetite		Dyspnoea: 53% vs 29% and 19% respectively	
			12/62 (19%) dyspnoea	20/62 (32%) malaise/lethargy		Rhonchi: 49% vs 22% and 21% respectively	
			13/62 (21%) rhonchi	19/62 (31%) vomiting		Wheezing: 28% vs 15% and 15% respectively	
			9/62 (15%) wheezing	4/62 (7%) diarrhoea		Breath rate ≥40/min: 61/86 (71%) vs 28/61 (48%) and 5/41 (12%) respectively	
			~ 12/62 (20%) rales/crackles	18/62 (29%) abdominal pain		Breath rate ≥50/min (tachypnoea): 40/86 (47%) vs 14/66 (21%) and 3/41 (7%) respectively	
			22/62 (36%) normal breath sounds	23/62 (37%) headache		Less common in <2 y compared with 2-4 y and children ≥5 y*:	
			13/62 (21%) decreased breath sounds	20/62 (32%) thoracic pain		Abdominal pain: 5% vs 21% and 29% respectively	
			12/41 (29%) breath rate ≥40/min (tachypnoea)			Headache: 3% vs 16% and 37% respectively	
			7/41 (17%) breath rate ≥50/min (tachypnoea)			Thoracic pain: 0% vs 6% and 32% respectively	
						Normal breath sounds: 19% vs 33% and 36% respectively	
						Decreased breath sounds: 7% vs 20% and 21% respectively	
Korppi et al, 2008 [8]	Udine, Italy	101 total	For children ≥5 y:	For children ≥5 y:	For children ≥5 y:	More common in <2 y and 2-4 y compared with children ≥5 y:	Weak
	Prospective enrolment of inpatients and outpatients with radiologically confirmed CAP with retrospective chart review of data (previously healthy children)	38 (38%) children ≥5 y old	34/38 (90%) cough	35/38 (92%) fever >37.5°C	26/38 (68%) alveolar infiltration		
			6/38 (16%) rhinitis	4/38 (11%) fever >39.5°C	3/38 (8%) pleural fluid	Looking ill 9/19 (47.4%) and 22/44 (50.0%) vs 9/38 (23.7%) respectively (*P* = 0.0381)	
			4/38 (11%) dyspnoea	11/38 (29%) chest pain			
			9/38 (24%†) tachypnoea‡	15/38 (40%) vomiting		Less common in <2 y and 2-4 y compared with children ≥5 y:	
			16/38 (42%†) crackles	8/38 (21%) refusal to eat			
			3/38 (8%†) dullness	5/38 (13%) headache		Chest pain: 0/19 (0.0%) and 4/44 (9.1%) vs 11/38 (28.9%) respectively (*P* = 0.0049)	
			26/38 (68%) decreased breath sounds	11/38 (29%) abdominal pain			
				9/38 (24%) looking ill		Vomiting: 1/19 (5.3%) and 14/44 (31.8%) vs 15/38 (39.5%) respectively (*P* = 0.0184)	
				1/38 (3%) chills			
Harris et al, 1998 [9]	Multiple centres in USA	420 total	For >5-16 y:	For >5-16 y:	Not specified	More common in ≤5 y compared with >5-16 y:**	Moderate
	Double-blind RCT to evaluate effectiveness of oral azithromycin vs “conventional” therapy§ for CAP. Outpatients with radiologic pneumonia finding + tachypnoea + fever/history of fever within 24 h/cough/WCC≥12000/mm^3^ /positive clinical chest findings (excluding chronic issues)	225 (54%)>5-16 y including 156 (69%) in the azithromycin arm and 69 (31%) receiving conventional therapy	212/225 (94%) abnormal respiratory rate‖	104/225 (46%) fever¶		Fever: 140/195 (72%) vs 104/225 (46%) *(P <* .005)	
			221/225 (98%) cough			Wheezes: 74/195 (38%) vs 59/225 (26%) (*P* < 0.001)	
			155/225 (69%) rales			Less common in ≤5 y compared with >5-16 y:**	
			59/225 (26%) wheezes			Rales: 109/195 (56%) vs 155/225 (69%) (*P* < 0.007)	
**Studies based on clinical pneumonia diagnosis with or without chest radiograph:**							
Udomittipong et al, 2011 [10]	Bangkok, Thailand	88 total	78/88 (89%) cough	74/88 (84%) fever ≥37.8°C	Not specified	Not specified	Weak
	Retrospective study of inpatients with a clinical diagnosis of pneumonia to compare non-H1N1 with H1N1 cases + prospectively follow-up PFTs (including chronic conditions)	88 (100%) 5-15 y	30/88 (34%) dyspnoea	29/88 (33%) muscle pain			
			59/88 (67%) rhinorrhoea	28/88 (32%) headache			
			37/88 (42%) sore throat	25/88 (28%) nausea-vomiting			
			48/88 (55%) injected pharynx	8/88 (9%) diarrhoea			
			3/88 (34%) injected tympanic membrane	4/88 (4.5%) joint pain			
			27/88 (31%) chest retraction				
Forgie et al, 1991 [11]	Fajara, The Gambia	74 total	For 5-9 y:	For 5-9 y:	For 5-9 y:	More common in 1-4 y compared with 5-9 y††:	Weak
	Prospective study of inpatients with clinical diagnosis of ALRI and obvious chest indrawing (information regarding comorbid conditions not specified)	10 (14%) 5-9 y	10/10 (100%) indrawing (study selected for this clinical feature)	0/10 (0%) inability to drink	9/10 (90%) abnormal CXR	Flaring: 50/64 (78%) vs 5/10 (50%)	
			5/10 (50%) flaring		7/10 (70%) lobar consolidation	Inability to drink: 14/64 (22%) vs 0/10 (0%)	
			5/10 (50%) bronchial breathing			Crepitations: 31/64 (48%) vs 1/10 (10%)	
			1/10 (10%) crepitations			Normal breath sounds: 10/64 (16%) vs 0/10 (0%)	
			6/10 (60%) diminished air entry			Less common in 1-4 y compared with 5-9 y††:	
			1/10 (10%) wheeze			Diminished air entry: 24/64 (38%) vs 6/10 (60%)	
			0/10 (0%) normal breath sounds				
			0/10 (0%) cyanosis				
Macpherson et al, 2019 [12]	Multicentre study in Kenya	1832 total	For 5-9 y:	For 5-9 y:	Not specified	Not specified	Moderate
	Retrospective study of inpatients with a clinician diagnosis of pneumonia at discharge or death (including comorbid conditions)	1467 (80%) 5-9 y	937/1216 (77%) respiratory rate >30/min (tachypnoea)	559/1321 (42%) temperature ≥38°C (fever)			
			24/1418 (2%) central cyanosis	108/1400 (8%) reduced consciousness			
			232/1394 (17%) grunting	235/1347 (17%) cannot eat or drink			
			58/1382 (4%) acidotic breathing	89/1411 (6%) severe pallor			
			46/1356 (3%) stridor	158/1411 (11%) mild/moderate pallor			
			743/1416 (52%) difficulty breathing	1164/1411 (82%) no pallor			
			189/1396 (14%) wheeze	ie, 247/1411 (18%) any pallor present			
			504/1387 (36%) crackles				
			609/1400 (44%) chest wall indrawing	212/1406 (15%) convulsions			
			151/661 (23%) oxygen saturation <90%				
Salih et al, 1994 [13]	Khartoum, Sudan	213 total	For 5-14 y:	For 5-14 y:	For 5-14 y:	More common in 12-59 mo compared with 5-14 y††:	Weak
	Prospective study of inpatients with ALRI based on clinical or radiological findings (excluding cases with measles but including other comorbid conditions, 11/24 (46%) of children aged 5-14 y were underweight‡‡)	24 (11%) 5-14 y	22/24 (92%) cough	21/24 (88%†) fever	18/23 (78%) abnormal CXR		
			2/24 (8%) cyanosis	2/24 (8%) feeding difficulties	4/23 (17%) lobal consolidation	Crackles (rales or crepitations): 88/92 (96%) vs 19/24 (79%)	
			11/24 (46%) nasal flaring			Less common in 12-59 mo compared with 5-14 y††:	
			18/24 (75%) chest recession				
			1/24 (4%) inspiratory stridor			Wheezes: 25/92 (27%) vs 11/24 (46%)	
			11/24 (46%) wheezes			Abnormal CXR: 57/89 (64%) vs 18/23 (78%)	
			19/24 (79%) crackles (rales or crepitations)			Lobal consolidation on CXR: 3/89 (3%) vs 4/23 (17%)	

CAP – community acquired pneumonia, RCT – randomised controlled trial, WCC – white cell count, PFTs – pulmonary function tests, ALRI – acute lower respiratory infection, CXR – chest x-ray, y – year, mo – months

*Values significant with *P* < 0.05 when the <2 years group was compared to the ≥2 years group (combined data for 2-4 years and ≥5 years).

†Corrected percentage value due to error in calculated percentage within study.

‡Tachypnoea was defined by age-specific WHO criteria: respiratory rate >50 breaths/min in infants <12 months old, >40 breaths/min in children aged 1-5 years and >30 breaths/min in children aged ≥6 years.

§Conventional therapy = amoxicillin/clavulanate if ≤5 years of age and erythromycin if >5 y of age.

‖Abnormal respiratory rate was defined as >24 breaths/min for patients ≤2 year of age and >20 breaths/min for patients >2 year of age.

¶Fever was defined as ≥100.5°F oral or ≥101°F rectal, or history in the last 24 h.

**Absolute numbers and percentages are extrapolated data.

††*P* values not calculated in this study.

‡‡Weight <80% of median value using National Center for Health Statistics reference values (United States Department of Health, Education and Welfare, 1976).

**Table 2:** Clinical features described in children aged 5-9 y diagnosed with pneumonia attributable to *Mycoplasma pneumoniae*

Authors, year	Study location, design & population	Patient numbers	Respiratory symptoms/signs	Extra-pulmonary symptoms/signs	Chest x-ray findings	Comparison with <5 y age group	EPHPP global rating score
**Studies based on radiological pneumonia diagnosis**							
Gao et al, 2015 [14]	Weifang, China	1933 patients with Mycoplasma pneumonia. 1249 patients with non-segmental/lobar Mycoplasma pneumoniae +684 patients with segmental/lobar Mycoplasma pneumoniae	For 4-6 y with segmental/lobar Mycoplasma pneumoniae:	For 4-6 y with segmental/lobar Mycoplasma pneumoniae:	Study selected those with segmental/lobar pattern on CXR:	More common in ≤3 y compared with 4-6 y and ≥7-14 y with segmental/lobar Mycoplasma pneumoniae:	Weak
	Retrospective study of inpatients with pneumonia defined by ICD-10 specifications, positive CXR findings* and positive Mycoplasma serology + PCR (excluding chronic conditions)		Cough	301/336 (90%) fever	For 4-6 y with segmental/lobar Mycoplasma pneumoniae:		
			Mean 9.34 ± 5.03 d	Mean 3.68 ± 4.64 d		Extra-pulmonary manifestations: 54/169 (32%) vs 88/336 (26%) and 37/179 (21%) respectively (*P* = 0.017)	
			90/336 (27%) gasping	88/336 (26%) extra-pulmonary manifestations	12/336 (4%) pleural effusion		
		336/684 (49%) 4-6 y	118/336 (35%) pulmonary crackles at onset	For ≥7-14 y with segmental/lobar Mycoplasma pneumoniae:	For ≥7-14 y with segmental/lobar Mycoplasma pneumoniae:	Less common in ≤3 y compared with 4-6 y and ≥7-14 y with segmental/lobar Mycoplasma pneumoniae:	
		179/684 (26%)≥7-14 y	For ≥7-14 y with segmental/lobar Mycoplasma pneumoniae:	168/179 (94%) fever	9/179 (5%) pleural effusion	Fever: 148/169 (88%) vs 301/336 (90%) and 168/179 (94%) respectively (*P* = 0.048)	
			Cough	Mean 5.25 ± 4.77 d			
			Mean 9.78 ± 7.23 d	37/179 (21%) extra-pulmonary manifestations			
			40/179 (22%) gasping				
			73/179 (41%) pulmonary crackles at onset				
Ma et al, 2015 [15]	Multicentre study in Taiwan	127 total	For >5 to <18 y:	For >5 - <18 y:	For >5 - <18 y:	More common in <5 y compared with >5 - <18 y:	Weak
	Prospective study of inpatients with radiographic evidence of CAP and positive Mycoplasma serology or PCR (including chronic conditions)	66 (52%)>5 - <18 y	64/66 (97%) cough	66/66 (100%) fever	~ 33/66 (50%)† lobar consolidation	Tachypnoea: 21/61 (34.4%) vs 8/66 (12.1%) (*P* = 0.003)	
			8/66 (12%) tachypnoea	Mean duration 7.94 ± 3.81 d	9/66 (14%) pleural effusion	Vomiting: 17/61 (27.9%) vs 9/66 (13.6%) (*P* = 0.005)	
				9/66 (14%) vomiting	0/66 (0%) pneumatocele	ICU Admission: 20/61 (32.8%) vs 8/66 (12.1%) (*P* = 0.006)	
				11/66 (17%) abdominal pain		O_2_ requirement: 29/61 (47.5%) vs 19/66 (28.8%) (*P* = 0.016)	
				8/66 (12%) diarrhoea		VATS: 9/61 (14.8%) vs 0/66 (0%) (*P* = 0.001)	
Youn et al, 2010 [16]	Daejeon, South Korea	191 total	For 6-14 y:	For ≥6-14 y:	For ≥6-14 y:	More common in ≤2 y and 3-5 y compared with ≥6-14 y:	Weak
	Retrospective study of inpatients with radiologically confirmed pneumonia and acute Mycoplasma infection reflected on serology testing at admission and discharge (information regarding comorbid conditions not specified)	81 (42%)≥6-14 y	81/81 (100%) cough	81/81 (100%) fever >38°C per axilla	25/81 (31%) bronchopneumonia	Bronchopneumonia: 23/29 (79%) and 48/81 (59%) vs 25/81 (31%) respectively (*P* < 0.001)	
			Data for other respiratory signs/symptoms not disaggregated by age	40/81 (49%) fever ≥7 d	56/81 (69%) segmental/lobar pneumonia	Less common in ≤2 y and 3-5 y compared with ≥6-14 y:	
				Duration of fever before admission 4.2 ± 2.3 d	33/81 (41%) mild segmental/lobar	Fever ≥7 d: 9/29 (31%) and 29/81 (36%) vs 40/81 (49%) respectively (*P* = 0.04)	
				Total duration of fever 6.1 ± 2.9 d	23/81 (28%) severe segmental/lobar	Segmental/lobar pneumonia: 6/29 (21%) and 33/81 (41%) vs 56/81 (69%) respectively (*P* < 0.001)	
				Duration of fever for severe segmental/lobar pneumonia (7.1 ± 2.6 d) longer than bronchopneumonia group (5.4 ± 2.8 d) (*P* < 0.05)			
				Data for other extra-pulmonary signs/symptoms not disaggregated by age			
Gordon et al, 2019 [17]	Jerusalem, Israel	353 total	For 6-18 y with CXR confirmed pneumonia:	For 6-18 y with CXR confirmed pneumonia:	For 6-18 y with CXR confirmed pneumonia:	Less common in <6 y compared with 6-18 y with CXR confirmed pneumonia:	Weak
	Retrospective study of inpatients with oropharyngeal swab positive for Mycoplasma on PCR testing (including chronic conditions)	172/353 (49%) 6-18 y	90/104 (87%) cough				
			17/104 (16%) sputum production	52/104 (50%) gastrointestinal symptoms	84/104 (81%) consolidation	Pharyngitis: 5/90 (6%) vs 18/104 (17%) (*P* = 0.018)	
			18/104 (17%) pharyngitis	18/104 (17%) headache	10/104 (10%) bilateral consolidation	Headache: 4/90 (4%) vs 18/104 (17%) (*P* = 0.014)	
			50/104 (48%) tachypnoea	91/104 (88%) fever >38°C	20/104 (19%) interstitial pattern		
			28/104 (27%) oxygen saturation <90%				
			77/104 (74%) any finding on lung auscultation				
**Studies based on clinical pneumonia diagnosis with or without chest radiograph:**							
Defilippi et al, 2008 [18]	Genoa, Italy	102 total	For 5 –<10 y:	For 5 –<10 y:	For 5 –<10 y:	More common in <5 y compared with 5 –<10 y‡,§:	Weak
	Prospective study of inpatients with clinical and/or radiological evidence of LRTI and Mycoplasma positive PCR (excluding chronic conditions)	42 (41%) 5–<10 y	12/42 (29%) wheezing	34/42 (81%) fever ≥38.0°C	34/38 (89%) consolidation		
			31/42 (74%) cough	2/42 (5%) diarrhoea	24/34 (71% unilateral consolidation	Dyspnoea: 18/39 (46.15%) vs 6/42 (14.28%) (*P* = 0.004)	
			6/42 (14%) dyspnoea	5/42 (12%) vomiting		Interstitial changes on CXR: 11/26 (42.31%) vs 2/38 (5.26%) (*P* < 0.0001)	
			5/42 (12%) coryza		10/34 (29%) bilateral consolidation	Less common in <5 y compared with 5 –<10 y§:	
			20/42 (48%) crackles		2/38 (5%) interstitial changes	Consolidation on CXR: 14/26 (53.85%) vs 34/38 (89.47%) (*P* = 0.004)	
Othman et al, 2005 [19]	Sydney, Australia	76 total	For 5-15 y:	For 5-15 y:	Data not disaggregated by age	More common in <5 y compared with 5-15 y:	Weak
	Retrospective study of hospital presentations/admissions with clinical and/or radiological features compatible with pneumonia and positive Mycoplasma serology (including comorbid conditions)	46 (61%) 5-15 y	11/46 (24%) coryza	20/46 (44%) lethargy		Coryza: 15/30 (50.0%) vs 11/46 (23.9%) (*P* = 0.019)	
			14/46 (30%) wheeze	15/46 (33%) vomiting		Vomiting: 17/30 (56.7%) vs 15/46 (32.6%) (*P* = 0.038)	
			15/46 (33%) breathlessness	1/46 (2%) diarrhoea		Diarrhoea: 11/30 (36.7%) vs 1/46 (2.2%) (*P* = 0.0001)	
			20/46 (44%) tachypnoea			Tachypnoea: 22/30 (73.3%) vs 20/46 (43.5%) (*P* = 0.023)	
			14/46 (30%) recession			Recession: 18/30 (60%) vs 14/46 (30.4%) (*P* = 0.016)	
			30/46 (65%) crackles				
Sondergaard et al, 2018 [20]	Hillerød, Denmark	134 total	For 7-15 y:	For 7-15 y:	For 7-15 y**:	Objective wheezing and cough (asthma-like symptoms): more common†† in <3 y compared to older children (*P* = 0.01)	Weak
	Retrospective study of inpatients with clinical and/or radiological features compatible with pneumonia and positive Mycoplasma serology or PCR (including chronic conditions)	88 (66%) 7-15 y	87/88 (99%) cough	79/88 (90%) fever ≥38°C	5% hilar adenopathy (exclusively)		
			14/88 (16%) wheezing	21/88 (24%) skin manifestation (all)¶	82% lobar infiltration		
			10/88 (11%) rhinorrhoea	11/88 (13%) urticarial rash	19% atelectasis	Children <2 y were admitted to the hospital earlier after the onset of symptoms than older children (*P* = 0.01)	
			23/88 (26%) sore throat	2/88 (2%) Stevens-Johnson Syndrome	9% pleural effusion		
			4/88 (5%) croup symptoms	26/88 (30%) nausea and/or vomiting	1/88 (1%) empyema		
			50/88 (57%) tachypnoea‖				
			20/88 (23%) auscultation – wheezing				
			50/88 (57%) auscultation – crackles/decreased breath sounds				

ICD-10 - International Classification of Diseases 10th edition, CXR – chest x-ray, PCR – polymerase chain reaction, CAP – community acquired pneumonia, LRTI – lower respiratory tract infection, ICU – intensive care unit, VATS – video-assisted thorascopic surgery, y – years, d – days

*Pneumonia pattern characterised by WHO Standardization of Interpretation of Chest Radiographs for the diagnosis of community acquired pneumonia in children.

†Study text states that “one-half of the children had lobar pneumonia in both groups”, however study Figure 3 suggests a higher number (between 40 and 60 patients with lobar pneumonia for each of the ≤5 years and >5 years groups).

‡Data for a symptom/sign was included if able to be disaggregated from combined data.

§*P* values relate to comparison of <5 years group with 5 to <10 years and 10-14 years groups.

‖Tachypnoea was defined as a respiratory rate >99^th^ percentile for age.

¶Includes any type of rash, urticaria and Stevens-Johnson Syndrome.

**112/134 total patients had CXRs, fraction of children aged 7-15 years who had CXRs not specified.

††Absolute numbers and percentages not described.

Three of the eight studies that explored all-cause pneumonia included patients with comorbid conditions, three specifically excluded those with comorbidities, and two did not specify information about comorbidities. A significant proportion of participants aged 5-9 years in study by Macpherson et al had comorbid disease including malaria (28.77%), asthma (10.91%), neurological disorders (10.77%), severe malnutrition (9.48%) or HIV (8.32%) [12]. Meanwhile, 46% of children aged 5-14 years in study by Salih et al were underweight [13], and a variety of underlying chronic comorbid conditions were described by Udomittipong et al but not disaggregated by age [10]. Within the group of studies addressing *Mycoplasma pneumoniae*, four included those with chronic conditions or comorbidities, two excluded children with these and one did not specify information about comorbidities. Chronic pulmonary disease and asthma were most frequently described as pre-existing underlying disease [17,19,20].

Most studies were of weak quality when assessed with the EPHPP tool (**Table 1** and **Table 2**). The exceptions were Macpherson et al [12] and Harris et al [9], which were assessed as moderate quality. There were seven retrospective observational studies, six studies with prospective recruitment of participants, one randomized controlled trial (RCT) and one descriptive study based on interview and questionnaire data. Describing clinical features of pneumonia was a primary objective in thirteen of the studies; two were not conducted with this as a primary aim but included clinical features of pneumonia in a description of participants. Many studies (8/15) did not specify or utilise a standardised data collection method. Although all studies included participants aged 5-9 years, study populations also included older and younger children. Three studies provided data disaggregated for the 5-9 age range exactly; the remaining twelve studies overlapped with the target population with a sufficiently close age range to be representative. In some studies, there was a paucity of disaggregated data relating to clinical features in children 5-9 years old. There were also differing definitions and terms for some clinical features between studies. Most importantly, the definition of fast breathing varied from >20 breaths per minute [9], to >40 breaths per minute [8], to a respiratory rate >99^th^ percentile for age [20].

### Study outcomes

Aggregated data regarding the proportion of older children with specific respiratory symptoms and extra-pulmonary clinical features is summarised in **Table 3**.

**Table 3:** Overall data regarding proportion of children with specific clinical features in included studies

	All-cause pneumonia				*Mycoplasma pneumoniae*			
	**Overall, mean (95% CI), range, (%)**	**Radiological diagnosis, mean (95% CI), (%)**	**Clinical diagnosis, mean (95% CI), (%)**	**Number of studies, number of total patients in whom feature was sought (n, n)**	**Overall, mean (95% CI), range, (%)**	**Radiological diagnosis, mean (95% CI), (%)**	**Clinical diagnosis, mean (95% CI), (%)**	**Number of studies, number of total patients in whom feature was sought (n, n)**
**Respiratory symptoms/signs:**								
Cough	89.7 (89.2-90.3)	89.4 (88.4-89.1)	90 (89.6-90.4)	Overall (5, 437),	91.4 (90.3-92.5)	94.5 (93.5-95.3)	86.3 (83.3-89.3)	Overall (5, 381),
	[R: 81-98]			Radiological (3, 112),	[R: 74-100]			Radiological (3, 251),
				Clinical (2, 325)				Clinical (2, 381)
Dyspnoea/difficulty breathing*	29.1 (28.2-30.8)	14.9 (13.7-16.1)	43 (42.3-43.7)	Overall (4, 1604),	23.1 (22.4-23.8)	24.6 (NA)	23.4 (20.7-26.1)	Overall (3, 603),
	[R: 11-52]			Radiological (2, 100),	[R: 14-33]			Radiological (1, 515),
				Clinical (2, 1504)				Clinical (2, 88)
Nasal flaring†	50 (46.7-53.3)	–	50 (46.7-53.3)	Overall (2, 34),	–	–	–	0
	[R: 46-50]			Clinical (2, 34)				
Grunting	17 (NA)	–	17 (NA)	Overall (1, 1394),	–	–	–	0
	[R: 17]			Clinical (1, 1394)				
Chest wall indrawing‡	50.0 (48.8-51.2)	–	50.0 (48.8-51.2)	Overall (3, 1512),	30.0 (NA)	30.0 (NA)	–	Overall (1, 46),
	[R: 31-75]			Clinical (3, 1512)	[R: 30]			Radiological (1, 46)
Tachypnoea§	55.4 (53.6-57.2)	48 (42.9-53.1)	77 (NA)	Overall (4, 1520),	40.1 (37.9-42.3)	8.5 (7.7-9.2)	50.1 (48.5-51.7)	Overall (4, 304),
	[R: 24-94]			Radiological (3, 304),	[R: 12-57]			Radiological (2, 170),
				Clinical (1, 1216)				Clinical (2, 134)
Hypoxia (oxygen saturation <90%)	23	–	23 (NA)	Overall (1, 661),	27 (NA)	27.0 (NA)		Overall (1, 104),
	[R: 23]			Clinical (1, 661)	[R: 27]			Radiological (1, 104)
Central cyanosis/cyanosis	3.3 (3.1-3.5)	–	3.3 (3.1-3.5)	Overall (3, 1452),	–	–	–	0
	[R: 0-8]			Clinical (3, 1452)				
Crackles‖	42.9 (41.6-44.2)	44.0 (42.4-45.6)	42 (41.2-43.8)	Overall (6, 1746),	51.2 (50.2-52.2)	40.8 (NA)	56.4 (53.8-59.0)	Overall (3, 603),
	[R: 10-79]			Radiological (3, 325),	[R: 37-65]			Radiological (1,515),
				Clinical (3, 1421)				Clinical (2, 113)
Wheeze	22.0 (21.3-22.7)	20.4 (19.4-21.4)	23.2 (22.2-24.2)	Overall (5, 1717),	25.0 (23.8-26.2)	–	25.0 (23.8-26.2)	Overall (3, 176)
	[R: 0-46]			Radiological (2, 287),	[R: 23-30]			Clinical (3, 176)
				Clinical (3, 1430)				
**Extra-pulmonary symptoms/signs:**								
Fever**	74.8 (73.6-76.0)	51.2 (46.5-56.9)	71.3 (70.0-72.6)	Overall (6, 1758),	91.7 (91.2-92.3)	94.2 (93.8-94.8)	85.4 (84.4-86.5)	Overall (6, 896)
	[R: 42-97]			Radiological (3, 325),	[R: 81-100]			Radiological (4, 766)
				Clinical (3, 1433)				Clinical (2, 130)
Headache	33.9 (31.8-36.0)	45.3 (43.4-47.2)	31.8 (NA)	Overall (4, 231),	17.0 (NA)	17.0	–	Overall (1, 104)
	[R: 13-54]			Radiological (3, 143),	[R: 17]			Radiological (1, 104)
				Clinical (1, 88)				
Reduced consciousness	8.0 (NA)	–	8.0 (NA)	Overall (1, 1400),	–	–	–	0
	[R: 8]			Clinical (1, 1400)				
Convulsions	15 (NA)	–	15 (NA)	Overall (1, 1406),	–	–	–	0
	[R: 15]			Clinical (1, 1406)				
Pallor††	18 (NA)	–	18 (NA)	Overall (1, 1411),	–	–	–	0
	[R: 18]			Clinical (1, 1411)				
Feeding difficulties‡‡	13.2 (12.7-13.7)	20.2 (20.0-20.4)	8.6 (8.1-9.1)	Overall (5, 1481),	–	–	–	0
	[R: 0-21]			Radiological (3, 100),				
				Clinical (2, 1381)				
Nausea/vomiting§§	32.8 (30.0-33.6)	35.1 (33.9-36.3)	28.4 (NA)	Overall (3, 188),	21.9 (20.6-23.1)	13.6 (NA)	24.7 (23.0-26.4)	Overall (4, 242),
	[R: 28-40]			Radiological (2, 100),	[R: 12-33]			Radiological (1, 66),
				Clinical (1, 88)				Clinical (3, 176)
Abdominal pain‖‖	29.0 (28.99-29.01)	29.0 (28.99-29.01)	–	Overall (2, 100),	17.0 (NA)	17.0 (NA)	–	Overall (1, 66),
	[R: 29]			Radiological (2, 100)	[R: 17]			Radiological (1, 66)
Chest pain‖‖,¶¶	30.6 (30.1-31.1)	30.6 (30.1-31.1)	–	Overall (2, 100),	–	–	–	0
	[R: 29-32]			Radiological (2, 100)				
Skin manifestation***	–	–	–	0	24.0 (NA)	24.0 (NA)	–	Overall (1, 88),
					[R: 24]			Radiological (1, 88)

*Includes dyspnoea/difficulty breathing/gasping/breathlessness, combined data from 4-6 and ≥7-14 age groups from Gao et al included [14].

†Includes flaring/nasal flaring.

‡Includes indrawing/recession/chest wall indrawing/chest recession/chest retraction, Forgie et al excluded from analysis as study selected for patients with indrawing [11].

§Includes all utilised definitions of tachypnoea and abnormal respiratory rate, data pertaining to respiratory rate of ≥40 breaths per minute rather than ≥50 breaths per minute included from Juvén et al [7].

‖Includes crepitations/rales/crackles/pulmonary crackles at onset, data included if able to be disaggregated from other abnormal breath sounds, combined data from 4-6 and ≥7-14 age groups from Gao et al included [14].

¶Includes wheeze/wheezes/wheezing/auscultation – wheezing, data included if able to be disaggregated from other abnormal breath sounds, fraction and percentage of children with auscultation finding rather than reported symptom included from Sondergaard et al [20].

**Includes all utilised definitions of fever, data pertaining to fever >37.5°C rather than fever >39.5°C included from Korppi et al [8].

††includes any pallor present

‡‡Includes inability to drink/poor appetite/refusal to eat/cannot eat or drink/feeding difficulties.

§§Data included if able to be disaggregated from other gastrointestinal symptoms.

‖‖Data included if able to be disaggregated from pain at other sites.

¶¶Includes chest pain/thoracic pain.

***Includes any type of rash, urticaria and Stevens-Johnson Syndrome.

Cough was the most common clinical feature, documented in around 90% of patients in both all-cause and *Mycoplasma* cohorts, whether diagnosed clinically or radiologically. Fever was also common in both cohorts but more common in *Mycoplasma* (91.7%, 95% confidence interval (CI) = 91.2-92.3) compared to all-cause pneumonia (74.8%, 95% CI = 73.6-76.0).

Tachypnoea was identified in around half of patients overall but less frequently in the *Mycoplasma* cohort (all-cause pneumonia 55.4%, 95% CI = 53.6-57.2 and *Mycoplasma pneumoniae* 40.1%, 95% CI = 37.9-42.3). The study of outpatients by Harris et al had the highest prevalence of tachypnoea but the lowest threshold for defining tachypnoea (>20 breaths per minute for children older than 2 years) [9]. The percentage of patients with tachypnoea was lower for patients with a radiological diagnosis (all-cause pneumonia 48.0%, 95% CI = 42.9-53.1 and *Mycoplasma pneumoniae* 8.5%, 95% CI = 7.7-9.2) compared to a clinical diagnosis (all-cause pneumonia 77.0% comprising 1 study with 937/1216 patients and *Mycoplasma pneumoniae* 50.1%, 95% CI = 48.5-51.7). Of note, less than 10% of patients with a radiological diagnosis of *Mycoplasma pneumoniae* had documented tachypnoea.

Dyspnoea/difficulty breathing was documented in 29.1% (95% CI = 28.2-30.8) of all-cause pneumonia patients and 23.1% (95% CI = 22.4-23.8) of *Mycoplasma pneumoniae* patients. In the all-cause pneumonia cohort, the proportion of patients with dyspnoea was higher in the clinical diagnosis group (43.0%, 95% CI = 42.3-43.7) compared to the radiological (14.9%, 95% CI = 13.7-16.1). Chest indrawing was observed in approximately half of all-cause pneumonia cases, all of which were based on clinical diagnosis. There was only one small study of *Mycoplasma pneumoniae* patients which documented chest-indrawing in 30.0% (14/46) of patients [19]. Crackles or crepitations were variably described between studies but documented in around one half of patients overall. Wheeze or rhonchi were described in around one quarter of patients.

Chest and abdominal pain were each included in two studies of all-cause pneumonia (radiological diagnosis) and both were documented in around one third of patients. Abdominal pain was included in one small study of *Mycoplasma pneumoniae* patients (radiological diagnosis) and was found in 17% (11/66) of patients [15]. Headache, nausea and vomiting also occurred in around one third of patients in the all-cause pneumonia cohort, though these are non-specific symptoms that may occur in a range of illnesses. Skin manifestations were described in one study addressing *Mycoplasma pneumoniae* with data disaggregated by age and, in this study, were found in 25% (21/88) children [20].

With respect to chest radiograph findings in all studies, one study by *Gao et al* selected for patients with segmental/lobar *Mycoplasma pneumoniae* and additionally reported on the presence of pleural effusions (4%-5%) [14]. Aside from this, only a small number of study participants overall in the 5-9 year age range had disaggregated chest radiograph findings reported (**Table 4**). Lobar changes were documented in around half of patients who had chest radiographs but any further conclusions are limited by the variable inclusion and diagnostic criteria and limited data.

**Table 4:** Chest radiograph findings document in studies in children 5-9 y with pneumonia

Chest radiograph findings*	All-cause pneumonia, mean (95% CI), range, (%)	Number of studies, number of patients, (n, n)	*Mycoplasma pneumoniae*, mean (95% CI), range, (%)	Number of studies, number of patients, (n, n)
Lobar/segmental pneumonia†	43.7 (31.0-57.4)	2, 33	59.6 (57.4 -61.8)	2, 147
	[R: 17-70]		[R: ~ 50-69]	
Interstitial changes‡	–	0	12.2 (10.6-13.8)	2, 142
			[R: 5-19]	
Pleural effusion/empyema§	17.9 (14.8-21.0)	2, 81	7.4 (7.0-7.8)	2, 581
	[R: 8-28]		[R: 4-14]	

*Data included if able to be disaggregated from other chest x-ray findings and both numerator and denominator clearly stated.

†Includes lobar consolidation/lobal consolidation/lobar infiltration and segmental/lobar pneumonia, *Gao et al* excluded from analysis as selected for patients with segmental/lobar pneumonia [14].

‡Includes interstitial changes/interstitial pattern.

§Includes pleural effusion/empyema, combined data from 4-6 y and ≥7-14 y groups from *Gao et al* included [14], data for pleural effusion rather than single case of empyema included from Sondergaard et al [20].

Outcome data for children aged 5-9 years with pneumonia were available from a single study of inpatients in Kenya, which was also the largest study in the review [12]. Macpherson et al described risk factors associated with mortality in children aged 5-14 years admitted to hospital with pneumonia [12]. Outcome information was available for 1825/1832 (99.5%) patients, of whom 145 (7.9%) died. Inpatient case fatality was higher in children aged 10-14 years compared to the 5-9 year age group (14.05% vs 6.43%, *P* < 0.001). For children aged 5-10 years, risk factors for death demonstrated in multi-variate analysis included the presence of severe pallor (OR = 9.89, 95% CI = 4.68 to 20.93, *P* < 0.001), mild/moderate pallor (OR = 2.85, 95% CI = 1.35-6, *P* < 0.006), reduced consciousness (OR = 6.27, 95% CI = 2.8-14.08, *P* < 0.001), central cyanosis (OR = 6.35, 95% CI = 1.33-30.25, *P* < 0.02), a weight for age Z-score of≤-3 SD (OR = 2.99, 95% CI = 1.61-5.55, *P* < 0.001) and comorbid HIV (OR = 2.49, 95% CI = 1.18-5.28, *P* < 0.017). A respiratory rate >30 breaths per minute and inability to drink were associated with poor outcome, though did not reach statistical significance. Sex, presence of grunting, crackles, chest wall indrawing and comorbid malaria were not associated with mortality and wheeze was found to be relatively protective (not statistically significant). Additional analysis demonstrated that the combination of clinical characteristics used by WHO to define severe pneumonia in children less than 5 years old was poor in discriminating those at risk of death (sensitivity: 0.56, specificity: 0.68 and AUC: 0.62) in this study.

Regarding pneumonia severity and the need for inpatient treatment in children aged 5-9 years, there is little additional data to draw upon beyond the study by Macpherson et al [12]. Studies involving outpatients either did not describe chest indrawing or did not disaggregate data by age in combination with admission status [8,9,19]. Whilst lethargy was documented frequently, reduced consciousness as a specific sign was only described in the study by Macpherson et al [12].

Comparison with clinical features of pneumonia in younger children was made in six out of eight all-cause pneumonia studies and all seven *Mycoplasma pneumoniae* studies (**Table 1** and **Table 2**). In all studies which included chest and abdominal pain and compared frequency between older and younger children, they were found to be more common in older children [6-8]. Crocker et al found that abdominal pain was a reported symptom in all 12 cases in which pleural effusion or empyema were detected in children aged 3-16 years [6]. Comparison of chest auscultation findings between age groups demonstrated no clear trends, with some studies finding crackles and wheeze to be more common in younger children but other studies reporting greater frequency in older children [7,9,13]. Similarly, one study found that normal breath sounds were more common in children older than 5 years and another found that it was less common [7,11]. Inconsistent use of terms for auscultation findings between studies limited comparison. In a study of 127 children with *Mycoplasma pneumoniae*, Ma et al found that children less than 5 years of age were more likely to have a severe illness course, including intensive care unit admission, supplemental oxygen requirement and need for video-assisted thoracoscopic surgery (VATS) [15]. Vomiting also occurred more often in younger children with *Mycoplasma pneumoniae* [15,19]. Segmental or lobar consolidation on chest radiograph was a more common finding in older children for both all-cause pneumonia and *Mycoplasma pneumoniae* groups [13,16,18].

Comparative analysis of clinical features between those with and without comorbidities was not possible as data was not disaggregated for subgroups of participants with comorbidities in the 5-9 year age range in studies that included such participants.

## DISCUSSION

There is a paucity of quality evidence describing clinical features of pneumonia in children aged 5-9 years. This review explored findings from 15 studies, eight addressing pneumonia of all causes and seven addressing pneumonia attributable to *Mycoplasma pneumoniae*. The lack of evidence highlights the urgent need for research to understand clinical features, treatment approaches and outcomes for children 5-9 years of age with pneumonia, which remains one of the highest causes of death in this age group globally [3]. However, the evidence that does exist indicates that applying existing WHO definitions of pneumonia for children under 5 years of age, to this older age group, is likely to lower the diagnostic yield.

Current WHO guidelines for children under 5 years old distinguish simple cough from pneumonia based on the presence or absence of tachypnoea. Among studies in this review, tachypnoea lacked standard definitions and this complicates interpretation of findings. However approximately only half of patients in the all-cause pneumonia cohort were documented to have tachypnoea, and this was lower for *Mycoplasma pneumoniae* patients, notably those diagnosed radiologically. Higher proportions of children with pneumonia in clinically diagnosed groups may represent later diagnosis. Alternatively, it may reflect greater emphasis on accurate measurement and recording of respiratory rate in clinicians using clinical diagnosis. The data on clinical diagnosis regarding tachypnoea in the all-cause pneumonia cohort is based on the Kenyan study, which is a cohort of sick children in a high burden setting. Yet, even amongst these patients around 1 in 4 did not have tachypnoea (respiratory rate >30 breaths per minute) documented on admission [12]. The measurement of respiratory rate is a skill which is often not performed well or documented correctly; the evidence indicates that it cannot be relied upon to identify pneumonia among older children with cough [21].

If tachypnoea cannot be relied on to diagnose pneumonia in older children, then addition of other symptoms to aid diagnostic approaches should be considered. Although the study numbers are small, chest pain and abdominal pain were relatively common in children aged5-9 years with all-cause pneumonia, whether due to their ability to report symptoms, or to the likelihood that researchers sought to identify these symptoms in older children. Chest radiographs may also have a greater role in diagnosing children with pneumonia in this age group, particularly in the setting of persistent cough and fever without other signs to confirm pneumonia (or alternative diagnoses). It should be noted, the data on chest radiograph findings in pneumonia in this age group is limited and there is insufficient data supporting the use of radiographs to distinguish pneumonia aetiology (eg, *Mycoplasma* from all-cause).

Symptoms used to define severe pneumonia in children <5 years of age, such as reduced conscious state, central cyanosis and/or hypoxia (oxygen saturation <90%) and inability to eat or drink [1,2], still have relevance in older children in low and lower-middle income settings in terms of their risk of mortality and therefore the severity of pneumonia. Similarly, nutritional status and underlying chronic conditions (including HIV) are associated with mortality in older children and should be part of any risk stratification approach used by clinicians to determine the need for admission and treatment [1,2]. Pallor, whether mild, moderate or severe, was identified as being associated with a higher risk of mortality in children 5-9 years old and should also be part of a clinician’s consideration of risk and patient disposition [12]. This is consistent with recent evidence suggesting that pallor is an important marker of serious disease in younger age groups [22-24]. The sign of chest indrawing has been an important and evolving marker of pneumonia severity and therefore need for admission in guidelines for children under 5 years old [25]. This review identified no data on the management of chest indrawing in children aged 5-9 years in the outpatient setting. Given chest compliance reduces with age [25], it is reasonable to suspect that chest indrawing may indicate greater severity in older children, as its presence may suggest generation of greater intrathoracic pressures to maintain ventilation. The Kenyan study in this review examined risk of death in older children with pneumonia and found no association between chest indrawing and mortality [12]. This finding, among others described above, is based on a single study in one context and should be interpreted with caution. Of note no radiological studies of all-cause pneumonia documented the presence or absence of chest indrawing in patients, despite its potential importance in guiding treatment.

Our review identified several studies relating to *Mycoplasma pneumoniae* in children 5-9 years of age mostly from high income countries, from which data has been reported separately to not unduly influence data on all-cause pneumonia, and to consider differences in clinical features. While *Mycoplasma pneumoniae* is important in pneumonia in older children, the emphasis on this organism in this review may represent bias on the part of researchers in considering it above other aetiologies. There is a clear need for more data on other potential aetiologies (eg, influenza), but particularly those relevant in the global context, such as HIV and tuberculosis.

Based on the available evidence for *Mycoplasma pneumoniae*, there are no respiratory clinical features that can distinguish it from pneumonia of other aetiologies in children aged 5-9 years. This is consistent with other studies that demonstrated no clinical or radiological features to identify *Mycoplasma pneumoniae* and guide therapeutic decisions [26,27]. Considering *Mycoplasma pneumoniae* as an aetiology and treating this possibility is therefore important, including in HIV positive children among whom it has also been shown to be common [28]. Skin symptoms may be useful in distinguishing *Mycoplasma pneumoniae* as a potential aetiological agent in pneumonia in older children, however there may be bias in seeking and reporting on these symptoms in studies focused on *Mycoplasma* *pneumoniae* and disaggregated supportive evidence was available from only one study in this review [20]. Separately, a review by Schalock and Dinulos [29] specifically addressing *Mycoplasma pneumoniae*-induced cutaneous disease in paediatric and adult populations and a study by Sauteur et al [30] in paediatric patients aged 3-18 years described skin manifestations as a feature of *Mycoplasma pneumoniae*, such as exanthematous skin eruptions, urticaria, erythema nodosum, *Mycoplasma pneumoniae*-induced rash and mucositis (MIRM) and Stevens-Johnson Syndrome. A key limitation in determining aetiology is that available diagnostic tests for *Mycoplasma pneumoniae* may not distinguish infection from carriage [31].

### Implications for WHO pneumonia guidelines

The relatively weak quality of studies and limited evidence in this review should be kept in mind when interpreting the findings. Evidence related to risk factors for death, for example, is derived from a single study of moderate quality. Different definitions (eg, for tachypnoea), different nomenclatures (eg, crepitations) and absence of documentation of key signs (eg, chest indrawing) should be noted. Nonetheless, there are some implications to be considered for WHO guidelines while further research is conducted and evidence is generated.

Cough and fever are common clinical features in pneumonia in children aged 5-9 years. However, tachypnoea, used to define pneumonia according to WHO criteria in children <5 years of age, may not be present in older children with pneumonia. Inclusion of chest pain and abdominal pain in diagnostic approaches for older children might expand recognition of pneumonia in this age group, especially if other signs are absent. Furthermore, chest radiographs may have greater importance for diagnosis. Clear definitions of tachypnoea are required for both clinical application and to standardise future research.

Symptoms reflecting severity of pneumonia in children <5 years of age (eg, reduced conscious state, hypoxia and inability to drink) have relevance in older children in low resource settings with respect to risk of mortality, and therefore severity of pneumonia. Separate to these markers of severe disease, other patient factors such as poor nutritional status, comorbid chronic conditions and pallor are associated with poor outcomes. As a result, they should be part of the clinician’s consideration of risk of a poor outcome for children aged 5-9 years with pneumonia, and inform decision making on patient disposition.

There is minimal data on chest indrawing in children aged 5-9 years, particularly its management in outpatient settings, to guide management recommendations. Without further evidence, it may be safest to recommend admission if chest indrawing is present.

Although there are differences in the proportions of patients with clinical features between the all-cause pneumonia and *Mycoplasma* cohorts, these cannot be used to distinguish pneumonia of different aetiologies in children aged 5-9 years on an individual level. Guidelines should account for causative agents other than pneumococcus and antibiotic recommendations should be altered accordingly. The addition of an antibiotic to cover for *Mycoplasma pneumoniae* (eg, macrolide) when treating pneumonia in this age group should be strongly considered, particularly in severe cases, in children with malnutrition and/or other co-morbidities, and when deterioration occurs on alternate therapy. Skin symptoms may be useful in distinguishing *Mycoplasma pneumoniae* as a potential aetiological agent in pneumonia in children aged 5-9 years, though there is limited evidence available and large potential for bias.

### Limitations

This review was conducted with a rigorous systematic approach, broad search strategy to capture relevant publications and methods to minimise risk of bias. It was limited by the databases that were searched, restriction of publications to the English language and unavailability of two full-text articles. Overall, the key limitation is the breadth and depth of existing research pertaining to pneumonia in children aged 5-9 years that is available to inform decision making.

Further studies exploring clinical features of pneumonia in children aged 5-9 years are warranted to strengthen evidence and understanding of the presentation of pneumonia in this age group. Studies using consistent definitions of clinical features and age ranges would enable aggregation of data and comparison between studies and settings. A wider range of studies in outpatient and inpatient settings, which identify clinical features associated with pneumonia severity and help to define critical values of concern for key signs, eg, tachypnoea, would better identify children at risk of poor outcomes. Conversely, understanding the prevalence of features such as chest indrawing in outpatient settings would aid in guiding safe management of children in the community.

Studies describing pneumonia aetiology and associated clinical features in children aged 5-9 years are needed to better inform antimicrobial choices, or clinical scenarios in which particular antimicrobial choices should be prioritised.

Studies should also explore the presentation of pneumonia in children aged 5-9 years with comorbid chronic conditions, given that this group is likely to be at higher risk of recurrent and more severe pneumonia.

## CONCLUSIONS

There is a lack of evidence describing clinical features of pneumonia in children aged 5-9 years highlighting an urgent need for further research to guide best practice. Despite the quality and quantity of data, there are some findings which should be considered in relation to whether existing WHO definitions of pneumonia in children less than 5 years of age can be applied to older children. Based on limited data fever and cough are common in this age group, but tachypnoea cannot be relied on for diagnosis. While waiting for better evidence, broader attention to features such as chest and abdominal pain, the role of chest radiographs for diagnosis in the absence of symptoms such as tachypnoea, and risk factors which may influence patient disposition (chest indrawing, pallor, nutritional status) warrants consideration by clinicians.

## Additional material


Online Supplementary Document
