# Supplementary material for: A systematic review of the clinical features of pneumonia in children aged 5-9 years: Implications for guidelines and research
Source: J Glob Health. 2022 Mar 26;12:10002. doi: 10.7189/jogh.12.10002 (PMC8943783; doi:10.7189/jogh.12.10002)
Supplement: Online Supplementary Document [file jogh-12-10002-s001.pdf]

APPENDIX S1

Search strategy example – MEDLINE via Ovid:

1	exp *Pneumonia/di
2	((lower-respiratory-tract adj3 infection*) or pneumonia or pneumonias or lung-inflammation* or lobitis or nonspecific-inflammatory-lung-disease* or peripneumonia or pleuropneumonia or pleuropneumonitis or pneumonic-lung* or pneumonic-pleurisy or pneumonic-pleuritis or pneumonitides or pneumonitis or pulmonal-inflammation* or pulmonary-inflammation* or pulmonic-inflammation*).tw,kf.
3	diagnosis, differential/ or early diagnosis/
4	Physical Examination/
5	Medical History Taking/
6	(clinical-presentation* or clinical-feature* or clinical-sign* or clinical-symptom* or clinical-syndrome*).tw,kf.
7	(syndrome* or symptom*).tw,kf.
8	(five-year-old* or five-years-old* or six-year-old* or six-years-old* or seven-year-old* or seven-years-old* or eight-year-old* or eight-years-old* or nine-year-old* or nine-years-old* or 5-year-old* or 5-years-old* or 6-year-old* or 6-years-old* or 7-year-old* or 7-years-old* or 8-year-old* or 8-years-old* or 9-year-old* or 9-years-old* or aged-five or aged-5 or aged-six or aged-6 or aged-seven or aged-7 or aged-eight or aged-8 or aged-nine or aged-9 or five-years-of-age or 5-years-of-age or six-years-of-age or 6-years-of-age or seven-years-of-age or 7-years-of-age or eight-years-of-age or 8-years-of-age or nine-years-of-age or 9-years-of-age).af.
9	(greater-than-4-years or greater-than-four-years or older-than-4-years or older-than-four-years or less-than-10-years or less-than-ten-years or younger-than-10-years or younger-than-ten-years or 5-9-years or 5-to-9-years or five-to-nine-years or aged-5-9 or aged-five-to-nine or aged-5-to-9 or five-years-or-older or older-than-five-years or 5-years-or-older or older-than-5-years or greater-than-5-years or greater-than-five-years).af.
10	(1 or 2) and (3 or 4 or 5 or 6 or 7) and (8 or 9)
11	limit 10 to english language

TABLE S1

EPHPP tool modifications:

**Study design and studies**	**EPHPP tool modifications**
Retrospective observational study<br>*Gao et al* [16]<br>*Gordon et al* [13]<br>*Macpherson et al* [6]<br>*Othman et al* [11]<br>*Sondergaard et al* [14]<br>*Udomittipong et al*[a] [12]<br>*Youn et al* [19]	Component B: Study Design<br>- “Was the study described as randomized?” and related questions not applicable<br>Component C: Confounders<br>- Modified to, “Were important differences between groups described and considered in analyses?” A strong rating was given to a study that considered age, sex, ethnicity and comorbidities (unless cases with comorbid conditions were excluded). A moderate rating w given to a study that considered age, sex and comorbidities (unless cases with comorbid conditions were excluded). A weak rating was given to a study that did not consider at leas age, sex and comorbidities (unless cases with comorbid conditions were excluded).<br>Component D: Blinding<br>- Not applicable (no rating given)<br>Component G: Intervention Integrity<br>- Not applicable<br>Component H: Analyses<br>- (Q4) not applicable
Prospective observational study<br>*Defilippi et al* [20]<br>*Forgie et al* [8]<br>*Juven et al* [18]<br>*Korppi et al* [10]<br>*Ma et al* [15]<br>*Salih et al* [7]<br>*Udomittipong et al*[a] [12]	Component B: Study Design<br>- “Was the study described as randomized?” and related questions not applicable<br>Component C: Confounders<br>- Modified to, “Were important differences between groups described and considered in analyses?” A strong rating was given to a study that considered age, sex, ethnicity and comorbidities (unless cases with comorbid conditions were excluded). A moderate rating w given to a study that considered age, sex and comorbidities (unless cases with comorbid conditions were excluded). A weak rating was given to a study that did not consider at leas age, sex and comorbidities (unless cases with comorbid conditions were excluded).<br>Component D: Blinding<br>- (Q1) modified to, “Was (were) the outcome assessor(s) aware of the research question?

<table>
<tr><td></td><td>
- If data collection was by chart review, (Q2) was not applicable. If information was collected directly from patients or carers, (Q2) was applicable.
- Scoring of this Component was as follows:
<table>
<tr><th>Q1</th><th>Q2</th><th>Rating</th></tr>
<tr><td rowspan="4">Yes</td><td>Yes</td><td>Weak</td></tr>
<tr><td>No</td><td>Moderate</td></tr>
<tr><td>Can’t tell</td><td>Weak</td></tr>
<tr><td>Not applicable</td><td>Moderate</td></tr>
<tr><td rowspan="4">No</td><td>Yes</td><td>Moderate</td></tr>
<tr><td>No</td><td>Strong</td></tr>
<tr><td>Can’t tell</td><td>Moderate</td></tr>
<tr><td>Not applicable</td><td>Strong</td></tr>
<tr><td rowspan="4">Can’t tell</td><td>Yes</td><td>Weak</td></tr>
<tr><td>No</td><td>Moderate</td></tr>
<tr><td>Can’t tell</td><td>Weak</td></tr>
<tr><td>Not applicable</td><td>Moderate</td></tr>
</table>
Component G: Intervention Integrity
- Not applicable
Component H: Analyses
- (Q4) not applicable
</td></tr>
<tr><td>Interview and questionnaire based descriptive study
Crocker et al [17]</td><td>
Component B: Study Design
- “Was the study described as randomized?” and related questions not applicable
Component C: Confounders
- Modified to, “Were important differences between groups described and considered in analyses?” A strong rating was given to a study that considered age, sex, ethnicity and comorbidities (unless cases with comorbid conditions were excluded). A moderate rating w given to a study that considered age, sex and comorbidities (unless cases with comorbid conditions were excluded). A weak rating was given to a study that did not consider at leas age, sex and comorbidities (unless cases with comorbid conditions were excluded).
Component D: Blinding
- Not applicable (no rating given)
Component G: Intervention Integrity
- Not applicable
Component H: Analyses
- (Q4) not applicable
</td></tr>
<tr><td>Randomised controlled trial
Harris et al [9]</td><td>No modifications to EPHPP tool made</td></tr>
</table>

[a] This study by *Udomittipong et al* included both retrospective and prospective components [12]

The global rating of papers remained unchanged:
1. Strong = no weak ratings
2. Moderate = one weak rating
3. Weak = two or more weak ratings

TABLE S2

PRISMA 2020 Checklist:

Section and Topic	Item #	Checklist item
**TITLE**		
Title	1	Identify the report as a systematic review.

Section and Topic	Item #	Checklist item
**ABSTRACT**		
Abstract	2	See the PRISMA 2020 for Abstracts checklist.
**INTRODUCTION**		
Rationale	3	Describe the rationale for the review in the context of existing knowledge.
Objectives	4	Provide an explicit statement of the objective(s) or question(s) the review addresses.
**METHODS**		
Eligibility criteria	5	Specify the inclusion and exclusion criteria for the review and how studies were grouped for the synthe
Information sources	6	Specify all databases, registers, websites, organisations, reference lists and other sources searched o date when each source was last searched or consulted.
Search strategy	7	Present the full search strategies for all databases, registers and websites, including any filters and lim
Selection process	8	Specify the methods used to decide whether a study met the inclusion criteria of the review, including and each report retrieved, whether they worked independently, and if applicable, details of automation
Data collection process	9	Specify the methods used to collect data from reports, including how many reviewers collected data fr independently, any processes for obtaining or confirming data from study investigators, and if applicab process.
Data items	10a	List and define all outcomes for which data were sought. Specify whether all results that were compati study were sought (e.g. for all measures, time points, analyses), and if not, the methods used to decid
	10b	List and define all other variables for which data were sought (e.g. participant and intervention charact assumptions made about any missing or unclear information.
Study risk of bias assessment	11	Specify the methods used to assess risk of bias in the included studies, including details of the tool(s) study and whether they worked independently, and if applicable, details of automation tools used in the
Effect measures	12	Specify for each outcome the effect measure(s) (e.g. risk ratio, mean difference) used in the synthesis
Synthesis methods	13a	Describe the processes used to decide which studies were eligible for each synthesis (e.g. tabulating t comparing against the planned groups for each synthesis (item #5)).
	13b	Describe any methods required to prepare the data for presentation or synthesis, such as handling of r conversions.
	13c	Describe any methods used to tabulate or visually display results of individual studies and syntheses.
	13d	Describe any methods used to synthesize results and provide a rationale for the choice(s). If meta-ana model(s), method(s) to identify the presence and extent of statistical heterogeneity, and software pack
	13e	Describe any methods used to explore possible causes of heterogeneity among study results (e.g. sub
	13f	Describe any sensitivity analyses conducted to assess robustness of the synthesized results.
Reporting bias assessment	14	Describe any methods used to assess risk of bias due to missing results in a synthesis (arising from re
Certainty assessment	15	Describe any methods used to assess certainty (or confidence) in the body of evidence for an outcome
**RESULTS**		
Study selection	16a	Describe the results of the search and selection process, from the number of records identified in the s the review, ideally using a flow diagram.
	16b	Cite studies that might appear to meet the inclusion criteria, but which were excluded, and explain why
Study characteristics	17	Cite each included study and present its characteristics.
Risk of bias in studies	18	Present assessments of risk of bias for each included study.
Results of individual studies	19	For all outcomes, present, for each study: (a) summary statistics for each group (where appropriate) a (e.g. confidence/credible interval), ideally using structured tables or plots.
Results of syntheses	20a	For each synthesis, briefly summarise the characteristics and risk of bias among contributing studies.
	20b	Present results of all statistical syntheses conducted. If meta-analysis was done, present for each the confidence/credible interval) and measures of statistical heterogeneity. If comparing groups, describe t

Section and Topic	Item #	Checklist item
	20c	Present results of all investigations of possible causes of heterogeneity among study results.
	20d	Present results of all sensitivity analyses conducted to assess the robustness of the synthesized resul
Reporting biases	21	Present assessments of risk of bias due to missing results (arising from reporting biases) for each syn
Certainty of evidence	22	Present assessments of certainty (or confidence) in the body of evidence for each outcome assessed.
**DISCUSSION**		
Discussion	23a	Provide a general interpretation of the results in the context of other evidence.
	23b	Discuss any limitations of the evidence included in the review.
	23c	Discuss any limitations of the review processes used.
	23d	Discuss implications of the results for practice, policy, and future research.
**OTHER INFORMATION**		
Registration and protocol	24a	Provide registration information for the review, including register name and registration number, or sta
	24b	Indicate where the review protocol can be accessed, or state that a protocol was not prepared.
	24c	Describe and explain any amendments to information provided at registration or in the protocol.
Support	25	Describe sources of financial or non-financial support for the review, and the role of the funders or spo
Competing interests	26	Declare any competing interests of review authors.
Availability of data, code and other materials	27	Report which of the following are publicly available and where they can be found: template data collec studies; data used for all analyses; analytic code; any other materials used in the review.
